# Incidence of Primary End Point Changes Among Active Cancer Phase 3 Randomized Clinical Trials

**DOI:** 10.1001/jamanetworkopen.2023.13819

**Published:** 2023-05-17

**Authors:** Marcus A. Florez, Joseph Abi Jaoude, Roshal R. Patel, Ramez Kouzy, Timothy A. Lin, Brian De, Esther J. Beck, Cullen M. Taniguchi, Bruce D. Minsky, Clifton D. Fuller, J. Jack Lee, Michael Kupferman, Kanwal P. Raghav, Michael J. Overman, Charles R. Thomas, Ethan B. Ludmir

**Affiliations:** 1 Department of Radiation Oncology, The University of Texas MD Anderson Cancer Center, Houston; 2 Program in Translational Biology and Molecular Medicine, Baylor College of Medicine, Houston, Texas; 3 Department of Radiation Oncology, The Johns Hopkins University School of Medicine, Baltimore, Maryland; 4 Department of Biostatistics, The University of Texas MD Anderson Cancer Center, Houston; 5 Department of Head and Neck Surgery, The University of Texas MD Anderson Cancer Center, Houston; 6 Department of Gastrointestinal Medical Oncology, The University of Texas MD Anderson Cancer Center, Houston; 7 Department of Radiation Oncology, Dartmouth Geisel School of Medicine, Norris Cotton Cancer Center, Lebanon, New Hampshire

## Abstract

**Importance:**

Primary end point (PEP) changes to an active clinical trial raise questions regarding trial quality and the risk of outcome reporting bias. It is unknown how the frequency and transparency of the reported changes depend on reporting method and whether the PEP changes are associated with trial positivity (ie, the trial met the prespecified statistical threshold for PEP positivity).

**Objectives:**

To assess the frequency of reported PEP changes in oncology randomized clinical trials (RCTs) and whether these changes are associated with trial positivity.

**Design, Setting, and Participants:**

This cross-sectional study used publicly available data for complete oncology phase 3 RCTs registered in ClinicalTrials.gov from inception through February 2020.

**Main Outcomes and Measures:**

The main outcome was change between the initial PEP and the final reported PEP, assessed using 3 methods: (1) history of tracked changes on ClinicalTrials.gov, (2) self-reported changes noted in the article, and (3) changes reported within the protocol, including all available protocol documents. Logistic regression analyses were performed to evaluate whether PEP changes were associated with US Food and Drug Administration approval or trial positivity.

**Results:**

Of 755 included trials, 145 (19.2%) had PEP changes found by at least 1 of the 3 detection methods. Of the 145 trials with PEP changes, 102 (70.3%) did not have PEP changes disclosed within the manuscript. There was significant variability in rates of PEP detection by each method (*χ^2^* = 72.1; *P* < .001). Across all methods, PEP changes were detected at higher rates when multiple versions of the protocol (47 of 148 [31.8%]) were available compared with 1 version (22 of 134 [16.4%]) or no protocol (76 of 473 [16.1%]) (*χ^2^* = 18.7; *P* < .001). Multivariable analysis demonstrated that PEP changes were associated with trial positivity (odds ratio, 1.86; 95% CI, 1.25-2.82; *P* = .003).

**Conclusions and Relevance:**

This cross-sectional study revealed substantial rates of PEP changes among active RCTs; PEP changes were markedly underreported in published articles and mostly occurred after reported study completion dates. Significant discrepancies in the rate of detected PEP changes call into question the role of increased protocol transparency and completeness in identifying key changes occurring in active trials.

## Key Points

**Question:**

What is the rate of primary end point (PEP) changes among active and ongoing cancer phase 3 randomized clinical trials?

**Findings:**

In this cross-sectional study of 755 cancer phase 3 randomized clinical trials, PEP changes after trial initiation were identified among 19% of trials. A total of 70% of trials did not have PEP changes reported in a published article.

**Meaning:**

The findings suggest that PEP changes after trial activation occur frequently and are underreported in trial articles.

## Teaser

This cross-sectional study assesses the frequency of reporting of primary end point changes among active oncology phase 3 randomized clinical trials.

## Introduction

Randomized clinical trials (RCTs) are pivotal to the advancement of clinical care and scientific decision-making. However, selective outcome reporting and alteration of end points undermine the validity, reproducibility, and clinical applicability of RCTs.^1^ Despite efforts to make protocols available^2^ and to require clinical trial registration,^3^ lack of transparency among RCTs remains a pressing concern.^4,5^ There has been considerable variability in reported PEP change rates due to inconsistencies and the limited scope in how PEP changes have been identified and detected.^1,5,6,7,8,9,10,11^ Most notably, it is unknown how often trials report changes in primary end points when publishing their work. We hypothesized that PEP changes among active cancer clinical trials occur at a substantial rate and are underreported in published articles. Herein, we provide, to our knowledge, the first comprehensive assessment of publicly available sources for documenting PEP reporting changes (ClinicalTrials.gov, published articles, and published protocols) among cancer clinical trials. We evaluated the incidence of and factors associated with PEP changes.

## Methods

**Figure 1.**
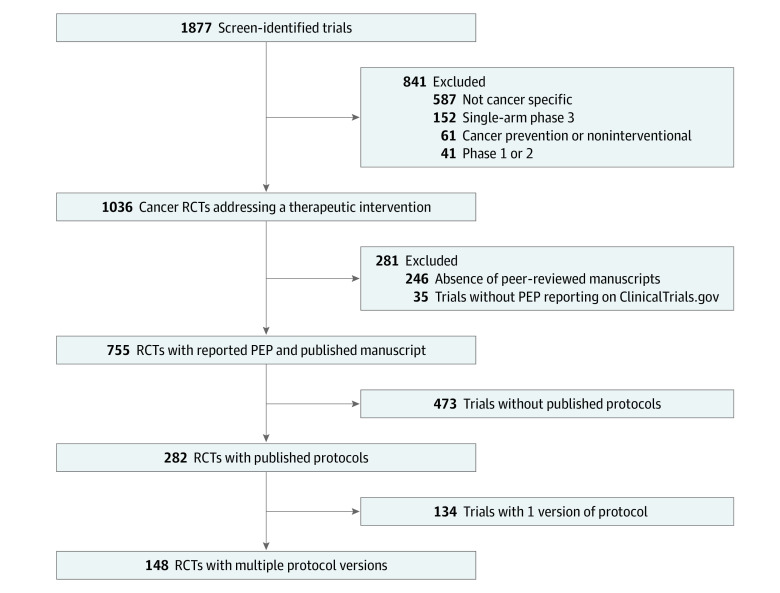
Flow Diagram of Clinical Trial Screening and Inclusion Criteria ClinicalTrials.gov was queried in February 2020 for all registered, cancer-specific, phase 3 randomized clinical trials (RCTs).

All data used for this cross-sectional study were collected from publicly available resources and did not require institutional review board approval per the Common Rule. The study followed the Strengthening the Reporting of Observational Studies in Epidemiology (STROBE) reporting guideline. We queried all completed phase 3, cancer-specific, interventional RCTs registered in ClinicalTrials.gov from inception through February 2020, with published articles reporting PEP results (Figure 1).^4^ In brief, trials were initially identified using ClinicalTrials.gov with the search parameters *cancer*, *all studies*, *phase 3*, and *with results*, excluding trials *not yet recruiting*. Published articles were identified using those linked to ClinicalTrials.gov and through PubMed searches using National Clinical Trial numbers. Trials were excluded if no PEP was provided or if any detected PEP change occurred before trial initiation. Changes in PEPs detected prior to trial initiation were not scored as PEP changes for the purposes of this study.

Primary end point changes were defined as previously described.^6,8^ These included (1) PEP reported as a secondary end point (SEP); (2) PEP omitted; (3) new PEP introduced, including an SEP reported as a PEP; and (4) change in the definition of the PEP, including changes in the timing of assessment of PEP, alteration of the population to be assessed for the PEP, changes in the method of PEP collection, and PEP criteria change. Minor changes in PEP wording or not clearly defined initial reporting were not considered a PEP change. Discrepancies in reported PEP were assessed using 3 reporting methods: (1) history of tracked changes on ClinicalTrials.gov, (2) self-reported changes noted in the article, and (3) changes reported within the protocol, including all available protocol documents (original and final versions of the protocol, protocol amendments, and summary of protocol changes, as available). Primary end point changes were determined by a single reviewer. Inconspicuous PEP changes were confirmed by 2 separate reviewers (J.A.J., E.B.L.).

### Statistical Analysis

Univariate and multivariable logistic regression analyses were performed to assess whether PEP changes, protocol availability, funding source, treatment location, treatment modality, or accrual goals were associated with US Food and Drug Administration approval or trial positivity, determined by whether the trial met the prespecified statistical threshold for PEP positivity. Statistical analyses were performed in Prism, version 9.1.2 (GraphPad) and RStudio, version 2021.09.0 (R Project for Statistical Computing). Two-sided *χ^2^* testing was used to compare PEPs across methods and protocol types at a significance level of *P* < .05.

## Results

A total of 1877 trials were identified on ClinicalTrials.gov. We identified 789 trials involving therapeutic interventions, with 755 of those trials reporting a PEP in a published article. One trial had a PEP change detected prior to trial initiation and was not included as having a PEP change in the present study. Of 755 trials meeting the inclusion criteria, 145 (19.2%) had detectable PEP changes occurring after the study start date by at least 1 of the 3 detection methods (ClinicalTrials.gov, article, published protocols) (Table 1). There was a significant difference in the detection rate of PEP changes among the methods (*χ^2^* = 72.1; *P* < .001). The highest rates of PEP changes were detected in published protocols (41 of 148 [27.7%]) followed by ClinicalTrials.gov (120 of 755 [15.9%]) and articles (43 of 755 [5.7%]) (*P* < .001) (Table 1). Of the 145 detected trials with PEP changes, 102 (70.3%) did not disclose changes within the article. The most common PEP changes observed were a PEP reported as an SEP (49 [33.8%]) and PEP definition changes (47 [32.4%]) (Table 1). There were no trials in which PEP changes were exclusively detected within the article. Of the 120 trials with changes that were observed on ClinicalTrials.gov, 63 (52.5%) had changes that occurred after the reported study primary completion date based on dates provided on ClinicalTrials.gov. Furthermore, using masking reporting on ClinicalTrials.gov, 85 of 145 trials with PEP changes (58.6%) were listed as open label compared with 309 of 610 trials without detected PEP changes (50.7%) (*P* = .052).

**Table 1.** Frequency and Type of PEP Changes Detected^a^

	Trials with PEP changes, No./total No. (%)
Detection method^b^	
Article	43/755 (5.7)
ClinicalTrials.gov	120/755 (15.9)
Protocol^c^	41/148 (27.7)
Total	145/755 (19.2)
Type of PEP changes	
≥1 PEP reported as SEP	49/145 (33.8)
≥1 PEP omitted	27/145 (18.6)
New PEP introduced	25/145 (17.2)
An SEP became a PEP	44/145 (30.3)
Definition of PEP changed	47/145 (32.4)
Other	1/145 (0.7)

Abbreviations: PEP, primary end point; SEP, secondary end point.

^a^ There were 145 trials with PEP changes, but PEP changes for some trials were identified using multiple detection methods and had multiple types of PEP changes.

^b^ *P* < .001, by Pearson χ^2^ method to compare PEP changes across methods of detection.

^c^ Of 282 trials with available protocols, 148 provided more than 1 version to allow for assessment of PEP changes.

**Figure 2.**
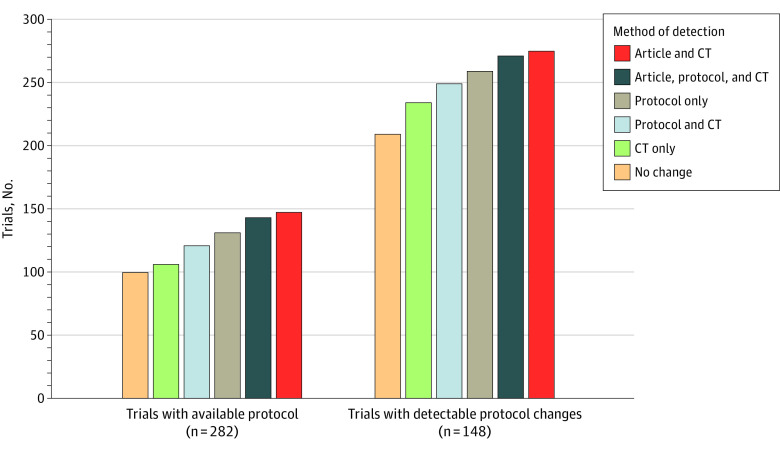
Number of Trials With Primary End Point (PEP) Changes by Method Of Detection CT indicates ClinicalTrials.gov.

Of 755 trials, 473 did not have available protocols; we observed PEP changes in 24 (5.1%) using the article and in 65 (13.7%) using CinicalTrials.gov (Table 2). Protocols were available for review for 282 of 755 of trials (37.4%). Among 69 trials with available protocols, only 12 (17.4%) had PEP changes consistently reported in the article, protocol, and ClinicalTrials.gov registration (Figure 2). Notably, 148 of 755 trials (19.6%) provided multiple versions of the protocol or a summary of protocol changes that allowed for assessment of protocol-level changes in PEPs, with the majority of these protocols (141 of 148 [95.3%]) providing a summary of changes. Higher rates of PEP changes were noted when multiple protocol versions were provided; PEP changes were observed among 47 of 148 trials with multiple versions of the protocol available (31.8%) compared with 22 of 134 trials providing 1 protocol version (16.4%) and 76 of 473 trials with no protocol (16.1%) (*χ^2^* = 18.7; *P* < .001) (Table 2).

**Table 2.** Protocol Availability for Trials in Which PEP Changes Were Detected

Trial protocol availability	Trials with PEP changes, No. (%)^a^				
	Article	ClinicalTrials.gov	Protocol	Total changes	No change
No protocol (n = 473)	24 (5.1)	65 (13.7)	0	76 (16.1)	397 (83.9)
1 Version (n = 134)	3 (2.2)	22 (16.4)	0	22 (16.4)	112 (83.6)
Multiple versions (n = 148)	16 (10.8)	33 (22.3)	41 (27.7)	47 (31.8)	101 (68.2)

Abbreviation: PEP, primary end point.

^a^ The denominator for percentages was the number for each type of protocol availability. *P* < .001, by χ^2^ test comparing total PEP changes for each protocol type with those for no changes of the same protocol type.

Of 145 trials with PEP changes, 89 (61.4%) were positive compared with only 309 of 610 trials without a PEP change (50.7%) (*P* = .01). Multivariable logistic regression analysis incorporating other trial-related factors demonstrated that PEP change remained independently associated with trial positivity (odds ratio, 1.86; 95% CI, 1.25-2.82; *P* = .003) (Table 3).

**Table 3.** Univariate and Multivariable Logistic Regression Analysis of Trial Positivity^a^

Variable	Univariate analysis		Multivariable analysis	
	OR (95% CI)	*P* value	OR (95% CI)	*P* value
Any PEP change	1.63 (1.12-2.40)	.01	1.86 (1.25-2.82)	.003
Accrual goal met	2.90 (1.97-4.33)	<.001	2.55 (1.71-3.85)	<.001
Cooperative group supported	0.42 (0.30-057)	<.001	0.59 (0.38-0.91)	.02
Industry funded	2.73 (1.91-3.93)	<.001	1.71 (1.06-2.77)	.03

Abbreviations: OR, odds ratio; PEP, primary end point.

^a^ Trial positivity was defined by whether the trial met the prespecified goal for significance at final analysis or publication based on reported trial goals (including the PEP) at the time of publication. Univariate and multivariable logistic regression was performed to assess whether PEP changes, accrual goals, and funding sources were associated with trial positivity.

## Discussion

To our knowledge, this is the first comprehensive analysis of PEP changes among phase 3 trials in clinical oncology that used 3 methods of detecting PEP changes. We found not only a high rate of PEP changes in clinical trials after trial initiation but also considerable variability in reporting of PEP changes across available methods. There was substantial underreporting of PEP changes within published articles, and only one-third of RCTs provided protocols. Furthermore, when assessing the association of PEP changes with trial masking, our data showed that more PEP changes occurred in trials that were listed as open label, whereas trials with no PEP change had similar numbers of trials that were listed as open label vs not open label. While ClinicalTrials.gov does not provide information regarding the masking of the sponsoring institution, the higher frequency of PEP changes in open-label trials suggests a need for greater transparency in reporting PEP changes.

Prior studies assessing rates of PEP change among multiple trials have reported variable rates of PEP discrepancies.^1,5,6,9,11^ Studies conducted on cancer-specific published trials found a PEP change of 12% when comparing protocols with published articles^5,11^ and 19% when comparing ClinicalTrials.gov with published articles.^11^ Another group identified a PEP change frequency of 6% when comparing registered PEPs with protocol PEPs.^9^ However, these prior reports each used a single detection method, compared end points among different methods, had small sample sizes, or limited the analysis to select journals. In the present study, we used all publicly available detection methods to both comprehensively quantify the PEP changes in cancer-specific RCTs in a major trial registry and differentiate among detection methods in detail.

There are multiple reasons for changes in PEPs. Masked review of data may reveal a high rate of discontinuation of therapy or slow accrual, which makes it difficult to properly assess the impact of a specific intervention. Prior to unmasking, results of a similar trial may also suggest that a different PEP may be more optimal in assessing the outcome of treatment.^12^ Furthermore, other ongoing trials may show toxic effects or other patient safety concerns, which would require changes in the study design and end points.^13^ Despite multiple potential reasons for altering study end points, our results showed a high rate of PEP changes detected, with few trials discussing those changes in published articles. It is possible that the changes in PEP reflected within the history of tracked changes on ClinicalTrials.gov were from clerical errors. This could also be true in comparing initial and final protocols, although less likely. Prior attempts by some of us to contact authors to learn about changes in PEPs have been largely unsuccessful.

These results highlight the need for increased transparency in reporting changes within trial protocols and within the article.^4^ Relying on authors to self-report PEP changes in published articles may be insufficient. While previous efforts have resulted in increased requirements for trialists to provide protocols as supplemental materials for published trial results,^2^ our data suggest that the current guidelines may be suboptimal to detect key changes in trial design and end points. Disclosure of substantial changes in PEPs, especially after trial initiation, should be required, as understanding the context and rationale for PEP choice (or alteration) is critical for trial interpretation and validity. This imperative is underscored by our finding that PEP changes appeared to be independently associated with trial positivity. Our findings suggest that trialists and journals should consider or require routine publication of protocols with a historical record of protocol changes in addition to mandating manuscript disclosure of any PEP changes that occurred after trial initiation. These policy changes would likely augment the transparency, validity, and interpretability of reported trial results.

### Limitations

Our analysis has several limitations. The results are based on only publicly available data; therefore, detectable changes relied on consistent and available reporting with each method. It is also not possible to know whether unintentional errors occurred during the initial reporting of PEPs on ClinicalTrials.gov that led to the discrepancies in PEPs observed in the study. Similar errors in reporting primary completion dates and changes on ClinicalTrials.gov may have led to our observation of PEP changes following the primary completion date. Studies have reported discrepancies in clinical trial registries related to listing of the principal investigators^14^ and reporting of results^10^ and registration dates.^15^ The variable quality of PEP descriptions and reporting on ClinicalTrials.gov that has been previously described may also have led to false-positive results or false-negative results in the present study, although we endeavored to mitigate this through the use of previously described definitions for major PEP changes.^6,8,11,16^ Furthermore, trials were not excluded if the initial PEP was not reported when the trial was first registered on ClinicalTrials.gov, and thus, we may have missed a small collection of early PEP changes. As discussed, there are numerous valid reasons to change the study design and end points prior to the study unmasking, including unexpected revelations related to study accrual, treatment-related toxic effects, and new scientific findings from similar studies. It is thus possible that some PEP changes were valid. However, based on available public data, we could not conclude whether the PEP changes were justified or detected due to unintentional clerical errors.

In addition, there could be multiple confounding variables related to the association between PEP change and trial positivity. Trials that justifiably change PEPs prior to unmasking and analysis because of internal or external factors may be more likely to have positive outcomes. Furthermore, trial positivity may be associated with variables not tested in the multivariable analysis, including statistical power or patient accrual. Although we could not fully elaborate on or determine the validity of PEP changes and its causal relationship to trial positivity, our study found a high rate of PEP changes that were not properly highlighted or justified.

## Conclusions

In this cross-sectional study, we detected a high rate of PEP changes among active cancer RCTs that occurred after trial initiation. Collectively, these PEP discrepancies were independently associated with trial positivity and were markedly underreported in published articles. The findings suggest that revision of journal policies to require trialists to publish and report PEP changes within the article and to provide protocols with multiple versions for reference is needed. The implications of PEP changes on trial outcomes and patient care need to be further elucidated.

## Notes

Supplement.
Data Sharing Statement
