# Supplementary material for: Incidence of Primary End Point Changes Among Active Cancer Phase 3 Randomized Clinical Trials
Source: JAMA Netw Open. 2023 May 17;6(5):e2313819. doi: 10.1001/jamanetworkopen.2023.13819 (PMC10193180; doi:10.1001/jamanetworkopen.2023.13819)
Supplement: Supplement. — Data Sharing Statement [file jamanetwopen-e2313819-s001.pdf]

Data Sharing Statement

Florez. Incidence of Primary End Point Changes Among Active Cancer Phase 3 Randomized Clinical Trials. *JAMA Netw Open*. Published May 17, 2023.
doi:10.1001/jamanetworkopen.2023.13819

Data
Data available: No

Additional Information
Explanation for why data not available: All data is already publically available
